# A survey to evaluate knowledge, perceptions and attitudes toward COVID-19 vaccinations among rheumatologists in Germany

**DOI:** 10.1007/s00296-021-04986-1

**Published:** 2021-09-08

**Authors:** Rebecca Hasseli, Alexander Pfeil, Andreas Krause, Hendrik Schulze-Koops, Ulf Müller-Ladner, Christof Specker, for the COVID-19 Task Force of the German Society for Rheumatology (DGRh), Bimba Hoyer, Hanns-Martin Lorenz, Anne Regierer, Jutta Richter, Tim Schmeiser, Anja Strangfeld, Reinhard Voll, Anna Voormann

**Affiliations:** 1 Department of Rheumatology and Clinical Immunology, Campus Kerckhoff, Justus-Liebig-University Giessen, Bad Nauheim, Germany; 2 Department of Internal Medicine III, University Hospital Jena, Jena, Germany; 3 Department of Rheumatology, Clinical Immunology and Osteology, Immanuel Hospital, Berlin, Germany; 4 Division of Rheumatology and Clinical Immunology, Department of Internal Medicine IV, University of Munich, Munich, Germany; 5 Department of Rheumatology and Clinical Immunology, Kliniken Essen-Mitte, Essen, Germany

**Keywords:** Immunmodulatory drugs, Rituximab, Inflammatory rheumatic diseases, Glucocorticoids, COVID-19 vaccines

## Abstract

The objective is to evaluate the attitude of rheumatologists regarding the use of COVID-19 vaccination in patients with inflammatory rheumatic diseases (IRDs). From February 2nd until March 15th, 2021, rheumatologists from Germany were asked to participate anonymously in a survey addressing their attitude with respect to COVID-19 vaccinations of IRD patients. The survey was completed by 214 participants (107 men, 103 women, 4 unspecified). More than half of the physicians (61%) were working in rheumatologic private practices and 62% had more than 20 years of experience in rheumatology. 90% reported to be at least confidential in handling issues of COVID-19 vaccination and 99% would recommend COVID-19 vaccination for IRD patients. The majority would not recommend to stop or reduce immunomodulatory drugs for vaccination except for rituximab. More than 70% would prefer vaccination with a mRNA vaccine for their IRD patients. This study shows that almost all rheumatologists in Germany support the COVID-19 vaccination for their IRD patients without reducing or terminating the actual immunomodulatory medication to potentially improve the response to the vaccine. This attitude is in accordance with the current recommendations of the German Society of Rheumatology regarding COVID-19 vaccination in IRD patients, and indicates that these have been well accepted and work in everyday clinical practice.

## Introduction

For more than 1 year, the world is struggling with the coronavirus SARS-CoV-2 disease (COVID-19). The available COVID-19 vaccines are offering light at the end of the pandemic tunnel by achieving a so-called ‘herd immunity’. Herd immunity works through achieving a threshold immunity at the population level that is able to interrupt the transmission chain of a given infectious disease, either by wild virus infection or by vaccination [1]. An optimal herd immunity is achieved by a high immediate and long-term vaccine efficacy [1]. For a vaccine with an efficacy of 95%, the required herd immunity level would be 63–76% [1]. In addition to a fundamental skepticism toward vaccinations, concerns prevail in Germany among patients and many physicians about the effectiveness and safety of vaccinations in inflammatory rheumatic diseases (IRDs), especially with respect to triggering flares. In addition, the so far unsurpassed rapid development of several new vaccines and the respective new technologies used to develop COVID-19 vaccines raised additional doubts and concerns in the population and among physicians [2].

Although until March 15th, 2021, no data on the safety and efficacy of the COVID-19 vaccines specifically in patients with IRDs were available, the Standing Committee on Vaccination (STIKO) of the Robert Koch Institute (RKI) recommends vaccination against COVID-19 of IRD patients [3]. The caveat, that live vaccines should not be administered in patients under immunosuppressive therapy, is not an issue for vaccination against COVID-19 since all currently licensed vaccines are non-live vaccines. Nevertheless, this has been and still is questioned repeatedly by patients and physicians.

To overcome the concerns and fears, both of the patients with IRD and their caring rheumatologists, the German Society for Rheumatology released the first recommendations for handling COVID-19 vaccinations in IRD patients in December 2020 [4, 5]. The recommendations were based also on the recommendations of the German National Authorities for vaccination strategies (Robert Koch Institute (RKI) and Paul Ehrlich Institute) and phase three clinical trials of the SARS-CoV-2 vaccines [3]. The main statements were that all available vaccines against COVID-19, including those using viral vectors (since they are non-replicating) are non-live vaccines and the use of nucleic acid technologies for the development of messenger RNA-vaccines does not imply threat or harm to the genetic integrity of the recipient [4, 6]. Therefore, these vaccines can and should be used without restriction in IRD patients, especially under immunosuppressive/immunomodulating therapy. Vaccinations should preferably be given when the IRD is in remission or a state of low activity. Moreover, to avoid disease flares, it is not recommended to reduce the dose or terminate immunomodulators in general [4]. In case of rituximab, if possible, an alternative treatment strategy should be considered to facilitate an adequate vaccination response without risking a flare [4]. However, the assumption to develop protective immune response upon vaccination was derived from other vaccines, as this aspect has not been evaluated specifically for COVID-19 vaccination. Similarly, derived from a markedly impaired immune response to established vaccinations during B cell depletion therapy, vaccination against COVID-19 is recommended not earlier than 4–6 months after the last application of rituximab [7–9]. Vice versa, the vaccination should be completed at least 2 weeks prior to the next rituximab infusion. The (so far unproven) idea to reduce immunosuppressive therapy in patients with IRD to facilitate or enhance the immune response upon vaccination was explicitly discouraged to avoid the risk of disease flares.

To increase the vaccination rate, rheumatologists should provide their patients with sufficient and consistent information. This study aimed to evaluate the attitude and knowledge of rheumatologists regarding the use of COVID-19 vaccination in patients with IRDs and thus examine the implementation of recommendations and identify any further needs.

## Methods

An online questionnaire with 21 questions, including baseline characteristics of the participants (e.g., age, work experience) and questions focusing on their attitude toward COVID-19 vaccination in IRD patients, was developed and announced to the German rheumatologists on a nationwide basis in a cross-sectional design using computer software SoSci Survey (version 3.1.06, Leiner [10], available at https://www.soscisurvey.de). The questions were designed as a selection of only one answer or selection of multiple answers. For pretest validation of the survey, a pilot test was run by a group of rheumatology staff members. Answers of the participating rheumatologists and health professionals were recorded by transferring the submitted data into an excel sheet. Only fully completed questionnaires were evaluated. Duplicate entries and incomplete questionnaires were excluded.

The study was open from February 2nd until March 15th, 2021. At this time, the nationwide vaccination campaign in Germany started with healthcare workers, especially healthcare workers involved in the treatment of COVID-19 patients, along with people aged 80 and over. Vaccination for IRD patients younger than 80 years were not available at that time period. Participants had to be specialized rheumatologists, trainees in clinical departments of rheumatology, or physicians treating mainly IRD patients. Participation was voluntary, anonymously, and without any reimbursement. Therefore, the ethics committee of the University of Giessen certified that no additional ethical approval was required for this study (January 26th, 2021) and the study was performed based on the rules of the ethics committee of the University of Giessen in accordance with the Declaration of Helsinki. Written informed consent was not required. This work was conducted in accordance to the recently published primer for author guidelines for reporting survey-based studies [11].

### Statistical analysis

The analysis was based on a descriptive statistic and additional exact binomial tests were used to assess differences between physicians’ characteristics and COVID-19-related answers using SPSS (version 26 IBM SPSS Statistics, Chicago, Illinois, USA). P values were 2-sided with a significance level set at *p* ≤ 0.05.

## Results

### Baseline characteristics (details see Table 1)

**Table 1:** Baseline characteristics of participating physicians

*Age (years)*						
< 30	31–40	41–50	51–60	61–70	> 70	No answer
7 (3.3%)	40 (18.7%)	45 (21%)	87 (40.7%)	29 (13.6%)	4 (1.9%)	2 (0.9%)
*Gender*						
Male					107 (50%)	
Female					103 (48.1%)	
Unspecified					2 (0.9%)	
No answer					2 (0.9%)	
*Specialty*						
Internal medicine and rheumatology					174 (81.3%)	
Internal medicine					6 (2.8%)	
Orthopedics					2 (0.9%)	
Orthopedics with specialization in rheumatology					2 (0.9%)	
Residency in rheumatology					20 (9.3%)	
Residency in internal medicine					5 (2.3%)	
Other					5 (2.3%)	

Workplace				
Private practice	Outpatient clinic	Hospital without teaching assignment	Academic teaching hospital	University hospital
130 (60.7%)	16 (7.5%)	7 (3.3%)	38 (17.8%)	41 (19.2%)
*Work experience (years)*				
< 5	5–10	> 10	> 20	> 30
17 (7.9%)	16 (7.5%)	48 (22.4%)	85 (39.7%)	48 (22.4%)

Involvement in teaching	Involvement in science
98 (45.8%)	126 (58.9%)

In total, 214 physicians (Table 1) from all federal German states participated and 90% were rheumatologists (specialists and residents). Most of them were middle aged with an equal distribution of males and females and had considerable work experience as rheumatologists (more than 80% between 11 and more than 30 years). Almost half of the participants were involved in teaching (46%) and/or science (59%). The majority of the physicians were working in private practices (61%), 37% in academic or university hospitals (Table 1).

### Physicians’ attitudes to vaccination against COVID-19 (details see Table 2)

**Table 2:** Questions regarding COVID-19 vaccination

Information sources for COVID-19 vaccination	
Training	154 (72%)
German Society for Rheumatology	179 (83.6%)
Robert Koch Institute	176 (82.2%)
Standing vaccination commission	159 (74.3%)
Online research	154 (72%)
Communication among colleagues	147 (68.7%)
General media (TV, radio)	131 (61.2%)
Association of panel doctors	76 (35.5%)

Self-assessment regarding handling COVID-19 vaccination and IRD				
Very uncertain	Uncertain	Undecided	Confident	Very confident
4 (1.9%)	4 (1.9%)	14 (6.5%)	136 (63.6%)	56 (26.2%)

How many patients are asking for further information about COVID-19 vaccination?			
No	< 50%	> 50%	Nearly every patient
3 (1.4%)	17 (7.9%)	55 (25.7%)	139 (65%)

Recommendation of COVID-19 vaccination for IRD patients	Self-vaccination against COVID-19
211 (98.6%)	211 (98.6%)

Under which dose of prednisolone (mg/day) would you recommend COVID-19 vaccination in IRD patients? (multiple selection possible)	
< 5 mg/day	116 (54.2%)
5–10 mg/day	121 (56.5%)
11–15 mg/day	40 (18.7%)
> 15 mg/day	15 (7%)
No influence of GC on recommendation for COVID-19 vaccination^a^	71 (33.2%)

Which type of COVID-19 vaccine do you prefer for IRD patients? (multiple selection possible)	
Moderna (COVID-19 Vaccine Moderna^®^)	153 (71.5%)
Biontech/Pfizer (Comirnaty^®^)	169 (79%)
AstraZeneca/Oxford (AZD1222^®^)	62 (29%)
Dead vaccine	2 (0.9%)
No opinion yet^a^	41 (19.2%)

Changes in therapy due to COVID-19 vaccination?						
	Pause before vaccination	Pause after vaccination	Pause before and after vaccination	Reduction of dose before vaccination	No change	No answer
csDMARDs	6 (2.8%)	26 (12.1%)	19 (8.9%)	3 (1.4%)	158 (73.8%)	2 (0.9%)
Azathioprine	3 (1.4%)	5 (2.3%)	17 (7.9%)	1 (0.5%)	183 (85.5%)	5 (2.3%)
Mycophenolate	4 (1.9%)	9 (4.2%)	20 (9.3%)	2 (0.9%)	165 (77.1%)	14 (6.5%)
TNF-inhibitors	4 (1.9%)	10 (4.7%)	18 (8.4%)	5 (2.3%)	172 (80.4%)	5 (2.3%)
IL-17-inhibitors	4 (1.9%)	10 (4.7%)	18 (8.4%)	4 (1.9%)	172 (80.4%)	6 (2.8%)
IL-6-inhibitors	5 (2.3%)	10 (4.7%)	22 (10.3%)	5 (2.3%)	166 (77.6%)	6 (2.8%)
IL-1-inhibitors	3 (1.4%)	10 (4.7%)	21 (9.8%)	5 (2.3%)	166 (77.6%)	9 (4.2%)
Abatacept	8 (3.7%)	13 (6.1%)	36 (16.8%)	5 (2.3%)	142 (66.4%)	10 (4.7%)
Rituximab	54 (25.2%)	7 (3.3%)	124 (57.9%)	6 (2.8%)	19 (8.9%)	4 (1.9%)
JAK-inhibitors	7 (3.3%)	14 (6.5%)	24 (11.2%)	1 (0.5%)	160 (74.8%)	8 (3.7%)

^a^Multiple selections were possible except for this item

Participants reported following the aforementioned recommendations of the German Society of Rheumatology (84%), recommendations of the Robert Koch Institute (82%) and its STIKO (74%). In addition, own online research (72%), online training (72%), personal communication among colleagues (69%), and public media [(television and radio) 61%] were used to stay informed. Only 35% of the participants reported informing themselves via the official association of panel doctors. The majority of physicians reported feeling at least confident with counseling their patients regarding vaccination (90%) and two-thirds that almost every patient spontaneously addressed questions regarding COVID-19 vaccination. Only 1.4% of the rheumatologists would not recommend COVID-19 vaccination for IRD patients. Half of the participants had already undergone vaccination themselves, 49% planned to do so and 1.4% stated that they would not get vaccinated.

### Rheumatology treatment strategy during vaccination (details see Table 2)

One-third of the participants reported that their recommendation for COVID-19 vaccination would be independent of the use of glucocorticoids (GC). More than half of the physicians would recommend vaccination at a maximum dose of 10 mg prednisolone daily, 19% up to a maximum dose of 15 mg prednisolone, and 7% even at higher doses. More than 80% reported checking the vaccination status of their patients routinely. More than 70% would recommend vaccination with Comirnaty^®^ (79%) and COVID-19 Vaccine Moderna^®^ (72%), while only 29% would recommend vaccination with Vaxzevria^®^ (AstraZeneca). At least 19% reported having no clear opinion about which type of vaccine to recommend.

More than 70% of the physicians would not recommend changing therapy with conventional synthetic (CS) disease-modifying anti-rheumatic drugs (DMARDs), and around 80% would recommend to continue TNF-, interleukin(IL)-1-, IL-6-, IL-17-, and JAK-inhibitors (Table 2). However, one-third would pause the therapy with abatacept, and only 9% would recommend vaccination during ongoing therapy with rituximab. Rituximab was considered to be rather paused before vaccination whereas abatacept was rather recommended to be withheld after vaccination.

Overall, the answers regarding the handling of immunotherapy and COVID-19 were independent of the gender of the respective physician, and whether the physicians worked in private practices or rheumatology departments.

## Discussion

To our knowledge, this study is the first to investigate the opinion of rheumatologists on COVID-19 vaccines in IRD patients and it illustrates the attitude of physicians toward the actually licensed vaccines in Germany. For IRD patients as well as for their rheumatologists, it is important to know if vaccination against COVID-19 will be effective, even under immunomodulating therapy, and does not induce disease flares. At the time of the survey, no data on the effectiveness and safety of COVID-19 vaccines in IRDs patients were available. The first paper to address this important issue was published at the end of March 2021 and covered selected immunosuppressants in a relatively small group of patients with chronic inflammatory diseases (mainly RA) [12]. Even though these primary results affirmed a sufficient immune response and no safety issues upon vaccination in IRDs under glucocorticoids (up to 15 mg daily), selected csDMARDs (mainly leflunomide) and biologics (mainly TNF-inhibitors), reliable data for the whole spectrum of IRDs and especially for many other immunomodulatory drugs are still lacking. Therefore, recommendations on vaccination against COVID-19, released by national and international societies rely on opinions of experts and adoptions from (even sparse) evidence from vaccination against other diseases [13]. The results of the survey indicate that more than 90% of the participating rheumatologists recommend vaccination against COVID-19 for their IRD patients, which is in line with the recommendation of the German society for rheumatology and STIKO [3, 5].

Despite the use of new platforms for development and lacking data in special diseases or under certain therapies, the administration of all available vaccines against COVID-19 is recommended for IRDs and also under immunosuppression by different official health institutions and rheumatological societies with some restrictions, mainly with respect to the use of rituximab. Concerns of potentially promoting disease flares through vaccination were outweighed unequivocally by the risk of COVID-19 in light of its pandemic nature.

The fact that the vast majority (99%) of German rheumatologists recommend their patients with IRDs to get vaccinated can be considered a success of the information policy of the German Society for Rheumatology. In a survey to explore the perspective of Egyptian Rheumatology staff members 70.1% agreed that they will recommend vaccination for IRD patients [14]. This is also reflected by the willingness of physicians to get vaccinated themselves (Table 2). The rate of physicians to get vaccinated themselves was higher compared to the results of a survey of healthcare workers in Turkey [15]. In this survey, only 68.4% of the physicians reported their willingness to get vaccinated against COVID-19 [15]. However, it is possible that more physicians with positive attitudes toward vaccines against COVID-19 completed the questionnaire, as participation was voluntary.

Regarding the use of different vaccines, the majority of participating rheumatologists in Germany prefers one of the mRNA vaccines for their IRD patients, only 29% would use the vector vaccine from AstraZeneca (Table 2). This is notable since the first warning due to reported cerebral sinus vein thromboses in the context of vaccination with the AstraZeneca vaccine came up in Germany shortly after closing our survey. There is no explanation for these results, as in the survey no further questions regarding the use of different vaccines were asked.

As immunomodulation might influence the response to vaccination, the question was if physicians would recommend to change treatment with immunomodulatory drugs with the intention to achieve a better immune response. On the contrary, discontinuation enhances the risk of disease flares, which might require the use of GC in higher doses. For pneumococcal vaccination, it could be observed that the use of prednisolone higher than 10 mg daily was associated with an impaired antibody response [16]. Therefore, physicians were asked, up to which dose of GC they would recommend COVID-19 vaccination in their IRD patients. In this survey, one-third reported that the use of GC would not have any impact on their advice for COVID-19 vaccination, 7% reported accepting dosages of more than 15 mg prednisone daily, 19% up to 15 mg, and most accepted doses of up to 10 mg (Table 2).

Compared to normal controls, a lower response to pneumococcal or influenza vaccines was reported for IRD patients under leflunomide, sulfasalazine, azathioprine, and hydroxychloroquine. However sufficient seroprotection was achieved in all cases [16–21]. This holds also true for the use of mycophenolate, of which immune response to vaccinations was investigated in organ-transplanted patients [21]. Likewise, under treatment with methotrexate, a moderately impaired humoral response to pneumococcal and influenza vaccines could be detected [21–25]. Discontinuation for up to 4 weeks after vaccination increased vaccine titres, however, such a long period off therapy might provoke disease flares [23, 25]. Three-quarters of the physicians reported not to change csDMARD therapy due to COVID-19 vaccination followed by 12% of the physicians who would recommend to pause csDMARD treatment for a while after vaccination. For practicability reasons, our survey did not differentiate between different csDMARDs.

For so-called targeted therapies, exact differentiation regarding the underlying mode of action was applied instead. Treatment with TNF-, IL-17-, and IL-6-inhibitors was not reported to be changed by the treating rheumatologists because of COVID-19 vaccination. This is justified by some studies revealing that immune response upon vaccination is not impaired under these treatments [17, 25–31]. Although to our knowledge, no data regarding IL-1 inhibition and immune response upon vaccinations are available yet, 78% of our physicians would not pause this around COVID-19 vaccination in IRD patients. In the survey of Egyptian Rheumatology staff members 71.7% of the participants reported to avoid treatment with biologics before vaccination [14]. This is contrary to our results, possibly due to national differences in the use of immunomodulation regarding COVID-19 vaccination or due to the availability of recommendations at this time.

For the use of abatacept, data are inconsistent regarding vaccination response. In a study of RA patients, subcutaneously administered abatacept with background csDMARDs did not alter an appropriate immune response to pneumococcal and influenza vaccines [32]. On the other hand, abatacept given intravenously (again with csDMARDs, mainly methotrexate) significantly reduced the humoral response to influenza A/H1N1 vaccine in RA patients during a pandemic in 2009 [33]. Therefore the ‘American College of Rheumatology Guidance for COVID-19 Vaccination in Patients with Rheumatic and Musculoskeletal Diseases’ recommends withhold of subcutaneous abatacept 1 week before and 1 week after the first vaccination dose but not for the second [34]. Even though this was not recommended in the German recommendations [5], 33% of rheumatologists in our survey advised pausing of abatacept around COVID-19 vaccination, the highest percentage of pausing among DMARDs except for rituximab. The latter was withhold by more than 90% of rheumatologists. Patients are advised to pause abatacept rather after than prior to vaccination, in contrast to the opposite way for rituximab. This is interesting, given the mode of action of these two compounds and the huge difference in biological half-time. Due to the known and markedly impairment of humoral responses to influenza and pneumococcal vaccines after administration of rituximab [22, 34–37], it is suggested performing vaccination against COVID-19 not earlier than 4–6 months after rituximab treatment [5, 8, 9].

Regarding JAK-inhibitors and vaccination responses, data are even more sparse. For tofacitinib a satisfactory response to the influenza vaccine could be observed, but response to pneumococcal vaccines was decreased [8, 38]. In this survey, three-quarters of the physicians reported not to change treatment with JAK-inhibitors due to COVID-19 vaccination, and if, this would rather be paused after than before vaccination (Table 2).

A limitation of our study is the absence of a control group, e.g., physicians treating other inflammatory diseases or general practitioners. Of the participating physicians, only 1.4% of rheumatologists would not recommend COVID-19 vaccination for IRD patients. The possible reasons for this decision were not further evaluated. As the survey was announced among members of the German society for Rheumatology, the results cannot be generalized to other specialities. As the sampling of our study was voluntary, the possibility of a selection bias (i.e., rheumatologists with special interest in vaccination or COVID-19) could not be eliminated. This survey was conducted during a single period of time during the third wave of the pandemic. Information, thoughts, decisions and perceptions may be a matter of change during the ongoing pandemic.

## Conclusions

Taken together, our study supports the idea that the timely recommendations of the German Society for Rheumatology regarding the handling of COVID-19 vaccination in patients with IRDs were well perceived by rheumatologists in Germany, providing high confidence in counseling their patients regarding COVID-19 vaccination.

## Data Availability

All data relevant to the study are included in the article.
